# Burnout and associated psychological issues among teachers: A Scoping Review

**DOI:** 10.1192/j.eurpsy.2023.2010

**Published:** 2023-07-19

**Authors:** B. Agyapong, G. Obuobi-Donkor, L. Burback, Y. Wei

**Affiliations:** Department of Psychiatry, University of Alberta, Edmonton, Canada

## Abstract

**Introduction:**

Worldwide, stress and burnout continue to be a problem among teachers, leading to anxiety and depression. Burnout may adversely affect teachers’ health and is a risk factor for poor physical and mental well-being. Determining the prevalence and correlates of stress, burnout, anxiety, and depression among teachers is essential for addressing this public health concern.

**Objectives:**

To determine the extent of the current literature on the prevalence and correlates of stress, burnout, anxiety, and depression among teachers

**Methods:**

This scoping review was performed using the PRISMA-ScR (Preferred Reporting Items for Systematic Reviews and Meta-Analyses extension for Scoping Reviews). Relevant search terms were used to determine the prevalence and correlates of teachers’ stress, burnout, anxiety, and depression. Articles were identified using MEDLINE (Medical Literature Analysis and Retrieval System Online), EMBASE (Excerpta Medica Data Base), APA PsycINFO, CINAHL Plus (Cumulative Index of Nursing and Allied Health Literature), Scopus Elsevier and ERIC (Education Resources Information Center). The articles were extracted, reviewed, collated, and thematically analyzed, and the results were summarized and reported.

**Results:**

When only clinically meaningful (moderate to severe) psychological conditions among teachers were considered, the prevalence of burnout ranged from 25.12% to 74%, stress ranged from 8.3% to 87.1%, anxiety ranged from 38% to 41.2% and depression ranged from 4% to 77%. The correlates of stress, burnout, anxiety, and depression identified in this review include socio-demographic factors such as sex, age, marital status, and school (organizational) and work-related factors including the years of teaching, class size, job satisfaction, and the subject taught.

**Conclusions:**

Teaching is challenging and yet one of the most rewarding professions, but several factors correlate with stress, burnout, anxiety, and depression among teachers. Highlighting these factors is the first step in recognizing the magnitude of the issues encountered by those in the teaching profession. Implementation of a school-based awareness and intervention program is crucial to resolve the early signs of teacher stress and burnout to avoid future deterioration.

**Disclosure of Interest:**

None Declared

